# 实性孤立性肺结节诊断模型的建立

**DOI:** 10.3779/j.issn.1009-3419.2016.10.12

**Published:** 2016-10-20

**Authors:** 微 喻, 波 叶, 力云 续, 兆宇 王, 涵波 乐, 善军 王, 捍波 曹, 振达 柴, 志军 陈, 清泉 罗, 永奎 张

**Affiliations:** 1 316021 舟山，温州医科大学附属舟山医院胸心外科 Department of Cardiothoracic Surgery, Afliated Zhoushan Hospital of Wenzhou Medical University, Zhoushan 316021, China; 2 200030 上海，上海交通大学附属胸科医院 Affiliated Chest Hospital of Shanghai Jiaotong University, Shanghai 200030, China; 3 316021 舟山，温州医科大学附属舟山医院肺癌研究中心 Lung Cancer Research Center, Affiliated Zhoushan Hospital of Wenzhou Medical University, Zhoushan 316021, China; 4 316021 舟山，温州医科大学附属舟山医院病理诊断中心 Pathology Diagnosis Center, Afliated Zhoushan Hospital of Wenzhou Medical University, Zhoushan 316021, China; 5 316021 舟山，温州医科大学附属舟山医院放射诊断中心 Radiology Diagnosis Center, Afliated Zhoushan Hospital of Wenzhou Medical University, Zhoushan 316021, China

**Keywords:** 孤立性肺结节, 预测模型, 独立预测因子, Solitary pulmonary nodules (SPNs), Prediction model, Independent predictors

## Abstract

**背景与目的:**

孤立性肺结节（solitary pulmonary nodule, SPN）是一个常见并富有挑战性的临床问题，其中尤以实性SPN为甚，本研究旨在建立实性SPN临床预测模型。

**方法:**

回顾性分析2015年1月-2015年12月上海胸科医院胸外科经手术病理证实的实性SPN患者317例（A组），分析其临床和计算机断层扫描（computed tomography, CT）影像特征：年龄、性别、吸烟史、肿瘤家族史、肿瘤既往史、结节直径、位置（上叶或者非上叶，左肺或者右肺）、边界清楚、边缘光滑、分叶征、毛刺征、血管集束征、胸膜牵拉征、空气支气管征、空泡征、空洞和钙化。通过单因素和多因素分析，寻找恶性实性SPN的独立预测因子，并建立临床预测模型。随后，利用温州医科大学附属舟山医院经手术病理证实的139例实性SPN患者作为B组，用于验证本模型的诊断效能，并绘制受试者工作特征曲线（receiver operating characteristic curve，ROC曲线）。

**结果:**

多因素*Logistic*回归分析筛选出年龄、肿瘤家族史、肿瘤既往史、边界清晰、分叶、毛刺、空气支气管征及钙化为恶性实性SPN患者的独立预测因子。利用筛选出的预测因子建立的诊断模型ROC曲线下面积为0.922（95%CI: 0.865-0.961），本模型的诊断准确率是84.89%，敏感性是90.41%，特异性是78.79%，阳性预测值是80.50%，阴性预测值是88.14%。

**结论:**

本研究建立的预测模型能较准确的诊断实性SPN，可为SPN患者的诊断提供有利帮助。

## Abstract (en)

**Background and objective:**

The solitary pulmonary nodule (SPN) is a common and challenging clinical problem, especially solid SPN. The object of this study was to explore the predictive factors of SPN appearing as pure solid with malignance and to establish a clinical prediction model of solid SPNs.

**Methods:**

We had a retrospective review of 317 solid SPNs (group A) having a final diagnosis in the department of thoracic surgery, Shanghai Chest Hospital from January 2015 to December 2015, and analyzed their clinical data and computed tomography (CT) images, including age, gender, smoking history, family history of cancer, previous cancer history, diameter of nodule, nodule location (upper lobe or non-upper lobe, left or right), clear border, smooth margin, lobulation, spiculation, vascular convergence, pleural retraction sign, air bronchogram sign, vocule sign, cavity and calcification. By using univariate and multivariate analysis, we found the independent predictors of malignancy of solid SPNs and subsequently established a clinical prediction model. Then, another 139 solid SPNs with a final diagnosis were chosen in department of Cardiothoracic Surgery, Affiliated Zhoushan Hospital of Wenzhou Medical University as group B, and used to verify the accuracy of the prediction model. Receiver-operating characteristic (ROC) curves were constructed using the prediction model.

**Results:**

Multivariate *Logistic* regression analysis was used to identify eight clinical characteristics (age, family history of cancer, previous cancer history, clear border, lobulation, spiculation, air bronchogram sign, calcification) as independent predictors of malignancy of in solid SPNs. The area under the ROC curve for our model (0.922; 95%CI: 0.865-0.961). In our model, diagnosis accuration rate was 84.89%. Sensitivity was 90.41%, and specificity was 78.79%, and positive predictive value was 80.50%, and negative predictive value was 88.14%.

**Conclusion:**

Our prediction model could accurately identify malignancy in patients with solid SPNs, thereby it can provide help for diagnosis of solid SPNs.

孤立性肺结节（solitary pulmonary nodule, SPN）通常是指肺内孤立存在的，圆形或者类圆形且直径≤3 cm的非透明病灶，该结节完全由肺实质所包绕，不伴有肺门或纵隔淋巴结肿大、肺不张及胸腔积液^[1]^。近年来，由于影像技术的不断发展以及低剂量螺旋计算机断层扫描（computed tomography, CT）的广泛使用，SPN的检出率呈明显的上升趋势。然而，SPN的正确诊断对于临床医生而言是一个巨大的挑战^[2, 3]^，其中，尤以实性SPN为甚。若实性SPN考虑恶性，因其恶性程度较高，应考虑进行积极的外科手术治疗，若考虑良性，可以避免一些不必要的外科治疗。国内外已有SPN良恶性预测模型的报道，但均是基于所有类型的SPN或者混杂密度SPN建立^[4, 5]^，而本研究将探讨建立预测实性SPN良恶性的诊断模型。

## 1 材料与方法

### 1.1 研究对象

回顾性分析上海交通大学附属胸科医院2015年1月-2015年12月期间经胸部CT检查发现且有手术病理证实的实性SPN患者317例。其中良性155例，恶性162例[平均（1.9±0.7）cm，T1aN0M0：16例；T1bN0M0：50例，T1bN1M0：5例，T1bN2M0：5例；T1cN0M0：58例，T1cN1M0：7例，T1cN2M0：13例；转移性8例]，男性161例，女性156例，年龄23岁-82岁，平均（57.0±10.7）岁，该组样本定义为A组，作为建立*Logistic*回归模型的训练样本。另外纳入温州医科大学附属舟山医院2013年1月-2014年12月期间经胸部CT检查发现且有手术病理证实的实性SPN患者139例，其中良性66例，恶性73例[平均（1.8±0.7）cm，T1aN0M0：8例；T1bN0M0：23例，T1bN1M0：3例，T1bN2M0：2例；T1cN0M0：27例，T1cN1M0：3例，T1cN2M0：7例]，男性73例，女性66例，年龄21岁-80岁，平均（58.2±10.3）岁，该组样本定义为B组，作为*Logistic*回归模型的验证样本。其中。所有良、恶性SPN患者的手术方式均为肺叶切除或者亚肺叶切除，病理结果由2名高年资病理医生明确诊断。

### 1.2 临床和CT影像资料

本研究收集的患者资料包括临床和CT影像资料，其中临床资料包括患者的年龄（岁）、性别、吸烟史、肿瘤家族史、肿瘤既往史（5年内有肺内、外肿瘤史者剔除）；另由2名高年资放射科医生收集CT影像资料，包括结节的直径、位置、边界清楚、边缘光滑、分叶征、毛刺征、血管集束征、胸膜牵拉征、空气支气管征、空泡征、空洞和钙化。

### 1.3 统计学方法

所有数据用SPSS 20.0统计软件进行处理，①单因素分析：符合正态分布、方差齐性的计量资料用Mean±SD表示，组间比较采用独立样本*t*检验；计数资料用频数和百分比表示，组间比较采用χ^2^检验。②多因素分析：对于单因素分析有统计学意义的因素，采用二元非条件*Logistic*回归分析筛选出恶性实性孤立性肺结节的独立预测因子，建立预测恶性实性SPN的数学诊断模型。应用受试者工作特征曲线（receiver operating characteristic curve, ROC），计算曲线下面积，确定最佳临界值，计算对应的敏感性，特异性，阳性预测值，阴性预测值。*P*＜0.05为差异有统计学意义。

## 2 结果

### 2.1 一般情况及病理诊断结果

本次研究调查的A组病例，实性SPN患者共计317例。其中，良性实性SPN患者155例，占48.90%，恶性实性SPN患者162例，占51.10%（表 1）。在B组，有66例实性SPN（47.5%）诊断为良性，73例（52.5%）诊断为恶性。两组患者性别（A：161男，156女*vs* B：73男，66女）、年龄（A: 57.0±10.7 *vs* B: 58.2±10.3）、结节直径（A: 1.8±0.7 *vs* B: 1.6±0.7）均无统计学意义（*P*＞0.05）。

**1:** A组317例实性SPN患者病理资料 Pathology data of 317 solid SPN patients in group A

Characteristic	Pathologic diagnosis	*n*
Benign		155 (48.90%)
	Tuberculoma	37
	Hamartoma	31
	Nonspecific inflammation	29
	Granulomatous inflammation	16
	Sclerosing alveolar cell tumor	12
	Inflammatory pseudotumor	8
	Aspergillus infection	8
	lymphnoditis	6
	Cryptococcal infection	5
	Glomus tumor	2
	Lung abscess	1
Malignant		162 (51.10%)
	Adenocarcinoma	130
	Squamous cell carcinoma	17
	Metastatic carcinoma	8
	Small-cell lung cancer	3
	Mucoepidermoid carcinoma	1
	Large-cell neuroendocrine carcinoma	1
	Large-cell lung cancer	1
	Plasmacytoma	1
SPN: solitary pulmonary nodule.		

### 2.2 实性SPN单因素和多因素分析

单因素分析显示，良性与恶性实性SPN患者在年龄、结节直径、肿瘤家族史、肿瘤既往史、边界清楚、边缘光滑、分叶征、毛刺征、胸膜牵拉征、空气支气管征、空泡征、钙化上均有统计学意义（*P*＜0.05）（表 2）。将此有统计学意义的因素作为自变量，通过二分类非条件*Logistic*回归进行分析，结果显示，年龄、肿瘤既往史、肿瘤家族史、边界清楚、分叶征、毛刺征、空气支气管征、钙化为恶性实性SPN的独立预测因素（表 3）。

**2:** A组患者临床和CT影像特征单因素分析 Univariate analysis of clinical and CT image characteristics of the patients in group A

Variable		Benign	Malignant	*P*
Age (yr)		54±11	60±9	< 0.01
Diameter (cm)		1.62±0.70	2.00±0.74	< 0.01
Gender	Male	77 (49.68%)	84 (51.85%)	0.699
	Female	78 (50.32%)	78 (48.15%)	
Smoking history	Yes	38 (24.52%)	50 (30.86%)	0.207
	No	117 (75.48%)	112 (69.14%)	
Family history of cancer	Yes	9 (5.81%)	33 (20.37%)	< 0.01
	No	146 (94.19%)	129 (79.63%)	
Previous cancer history	Yes	15 (9.68%)	42 (25.93%)	< 0.01
	No	140 (90.32%)	120 (74.07%)	
Position (1)	Upper lobe	72 (46.45%)	76 (46.91%)	0.934
	None-upper lobe	83 (53.55%)	86 (53.09%)	
Position (2)	Left side	66 (42.58%)	60 (37.04%)	0.313
	Right side	89 (57.42%)	102 (62.96%)	
Clear border	Yes	122 (78.71%)	158 (97.53%)	< 0.01
	No	33 (21.29%)	4 (2.47%)	
Smooth edge	Yes	42 (27.10%)	8 (4.94%)	< 0.01
	No	113 (72.90%)	154 (95.06%)	
Lobulation	Yes	91 (58.71%)	144 (88.89%)	< 0.01
	No	64 (41.29%)	18 (11.11%)	
Spiculation	Yes	39 (25.16%)	110 (67.90%)	< 0.01
	No	116 (74.84%)	52 (32.10%)	
Vascular convergence	Yes	7 (4.52%)	9 (5.56%)	0.673
	No	148 (95.48%)	153 (94.44%)	
Pleural retraction sign	Yes	48 (30.97%)	73 (45.06%)	0.010
	No	107 (69.03%)	89 (54.94%)	
Air bronchogram	Yes	11 (7.10%)	57 (35.19%)	< 0.01
	No	144 (92.90%)	105 (64.81%)	
Vocule sign	Yes	15 (9.68%)	40 (24.69%)	< 0.01
	No	140 (90.32%)	122 (75.31%)	
Cavity	Yes	5 (3.23%)	5 (3.09%)	0.960
	No	150 (97.77%)	157 (96.91%)	
Calcification	Yes	15 (9.68%)	5 (3.09%)	0.022
	No	140 (90.32%)	157 (96.91%)	
CT: computed tomography.				

**3:** 多因素*Logistic*回归分析结果 Result of multivariate *Logistic* regression analysis

Factor	B	S.E	Wals	*P*	OR	95%CI
Age	0.065	0.018	12.382	< 0.001	1.067	1.029-1.106
Family history of cancer	2.286	0.598	14.614	< 0.001	9.836	3.046-31.759
Previous cancer history	1.142	0.497	5.291	0.021	3.134	1.184-8.296
Clear border	4.048	0.791	26.207	< 0.001	57.260	12.158-269.685
Lobulation	1.342	0.383	12.290	< 0.001	3.826	1.807-8.100
Spiculation	2.192	0.347	39.867	< 0.001	8.952	4.533-17.676
Air bronchogram	1.589	0.468	11.509	0.001	4.900	1.956-12.272
Calcification	-2.007	0.718	7.806	0.005	0.134	0.033-0.549
Constant	-9.976	1.481	45.392	-	-	-
B: Regression coefficient; S.E: Standard error; OR: Odds ratio; CI: confidence interval.						

### 2.3 *Logistic*回归模型的建立

恶性实性SPN的预测模型：*P*=e^x^/(1+e^x^)，X=-9.976+（0.065×年龄）+（2.286×肿瘤家族史）+（1.142×肿瘤既往史）+（4.408×边界清楚）+（1.342×分叶征）+（2.192×毛刺征）+（1.589×空气支气管征）-（2.007×钙化）。公式中e为自然对数，若患者有肿瘤既往史，肿瘤家族史，结节边界清楚，有分叶征，毛刺征，空气支气管征，钙化，则用1表示，否则为0。

### 2.4

B组139例实性SPN患者的相关临床和CT影像特征代入本模型，诊断准确率是84.89%，并绘制ROC曲线（图 1）。ROC曲线下面积为0.922（95%CI: 0.865-0.961），最佳临界值*P*=0.402, 3对应的诊断敏感性为90.41%，特异性为78.79%，阳性预测值为80.50%，阴性预测值为88.14%。

**1:**
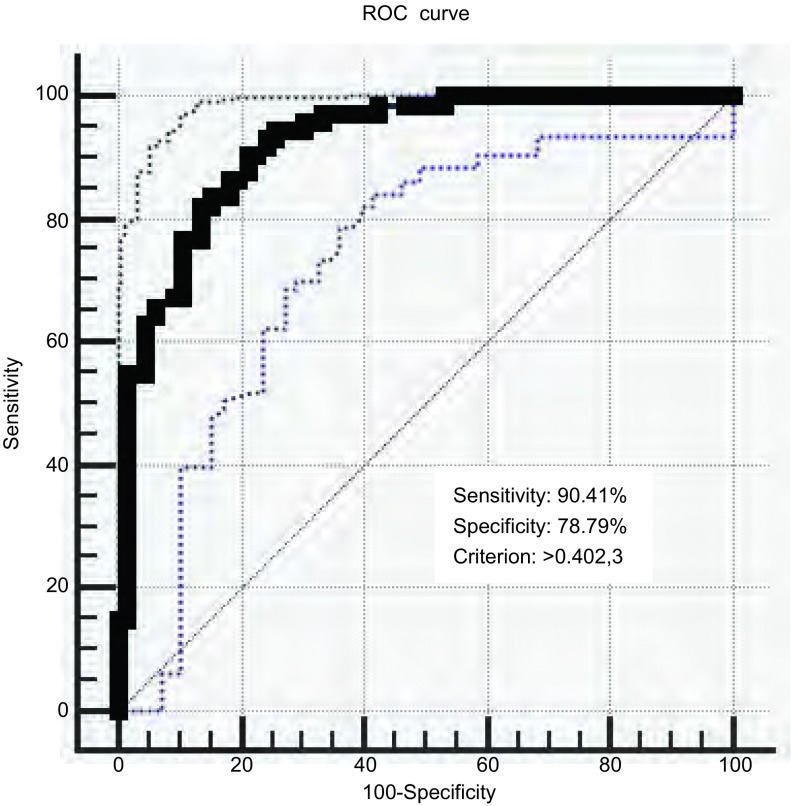
本模型ROC曲线下面积为0.922（图中实线），95%CI: 0.865-0.961（图中虚线），最佳临界值*P*=0.402,3对应的诊断敏感性为90.41%，特异性为78.79%。 The area under ROC curve of our model is 0.922 (solid line), 95%CI (confidence interval) is 0.865-0.961 (dotted line). An appropriate cut off point was selected at *P*=0.402,3, and the model achieved a sensitivity of 90.41%, a specificity of 78.79%. ROC: Receiver-operating characteristic.

## 3 讨论

肺癌是全球发病率和死亡率最高的恶性肿瘤之一，每年估计有180万新发病例，是癌症相关死亡中最常见的原因^[6]^。近年来，随着医学影像学技术和设备的不断发展，尤其是多层螺旋CT的广泛应用，SPN检出率显著增加，且SPN的良、恶性判断是临床工作的难点和热点之一^[7]^。所有SPN患者中，恶性SPN占40%^[8, 9]^，这些恶性SPN若能及时早期诊断和治疗可极大的提高肺癌患者的总体生存率和预后。以往，对于SPN患者的鉴别诊断主要基于经验性判断，这和临床医生、放射科医生的理论水平、实践经验和诊断能力密切相关。为了减少人为因素、提高诊断准确率，已有学者结合临床、CT影像特征建立了预测SPN良恶性的数学诊断模型^[10, 11]^，这些模型能引导临床医生和放射科医生选择合适的下一步诊疗计划。然而，之前建立的数学诊断模型，都是基于所有3种类型结节（纯磨玻璃结节、混杂磨玻璃结节、实性结节）或者混杂磨玻璃结节的数学诊断模型，而未见基于实性SPN的数学诊断模型。若实性SPN表现为恶性，其恶性程度明显高于其他类型的恶性肺结节，且进展迅速，需尽早作出明确诊断，及时进行外科手术治疗。

已经报道的模型中，如北京大学人民医院（Peking University People’s, PKUPH）模型、Mayo模型、Veterans Affairs模型以及McWilliams等^[12, 13]^报道的模型，均为应用于所有类型的SPN。本研究收集了实性SPN相关临床和CT影像特征，建立了基于实性SPN的数学诊断模型。通过多因素*Logistic*回归分析，筛选出年龄、肿瘤既往史、肿瘤家族史、边界清楚、分叶征、毛刺征、空气支气管征、钙化为恶性实性SPN的独立预测因子。已有报道，吸烟史和恶性肺结节之间的关系，如Mayo模型，Veterans Affairs模型筛选出吸烟史是预测恶性SPN的独立预测因子，而本研究显示，吸烟史不是恶性SPN的独立预测因子，可能的原因是我们的A组恶性病例，与其他模型相比，腺癌患者比例更高，而腺癌相对于其他几种类型肺癌，与吸烟史的联系并不紧密。Mayo模型显示，孤立性肺结节的位置是恶性的一项独立相关因素，恶性SPN在右肺和上叶更多见^[14]^。但本模型未见其相关性，可能因本研究入组的良性病例中结核比例较大，而结核好发于上叶，造成了良恶性肺结节患者中结节位置差异并不明显。结核在国内的发生率高于西方国家，故认为结节位置与实性SPN恶性概率不存在统计学关系是可以接受的。已有学者研究显示，边界清楚高度提示为良性结节，结节边界清楚，SPN的恶性可能性会降低75%^[15]^。而我们的研究显示边界清楚为恶性实性SPN的独立预测因子。可能的原因是本研究纳入的良性患者里非特异性炎性结节比例偏高，而非特异性炎性结节的边界是模糊的。分叶征是受到患者肿瘤生长不均、分化不一等影响，是恶性肺结节的典型表现，而良性结节的分叶征不明显，相对比较少见^[16]^。与本研究结果相符合，分叶征是恶性肺结节的危险因素，分叶征的出现高度提示肺结节为恶性。空气支气管征在病理上是一种形态正常或异常的细支气管，常有扭曲，扩张、变尖或中断，可为单支或多支，在走形上无一定的规律性^[17]^，本研究显示，空气支气管征提示结节为恶性。本研究的最大特点是我们纳入的病例都是实性SPN，对于该类诊断较困难的结节，收集了上海交通大学附属胸科医院和温州医科大学附属舟山医院的实性SPN病例，样本量较大，而且，应用上海交通大学附属胸科医院病例建立的数学诊断模型，采用温州医科大学附属舟山医院的病例进行外部验证时，诊断准确率较高，说明本研究建立的模型适用性较好。B组139例实性SPN患者的相关临床和CT影像特征代入本模型绘制的ROC曲线下面积为0.922（95%CI: 0.865-0.961），最佳临界值*P*=0.402, 3对应的诊断敏感性为90.41%，特异性为78.79%，阳性预测值为80.50%，阴性预测值为88.14%。

本研究的局限性在于模型的建立是基于回顾性研究，且仅限于两个单位，将来需要多中心更大样本量的进一步深入研究。模型不够简化，临床医师使用起来较复杂，尚不能完全代替临床医生的经验性判断，其作为一种临床估计工具，不能代替作为金标准的病理诊断。由于原始数据所限，未纳入肿瘤标记物，如：癌胚抗原（carcinoembryonic antigen, CEA）、细胞角蛋白19的可溶性片（cytokeratin-19 fragment, Cyfra21-1）、血浆蛋白等实验室检查，可能会让本模型的诊断准确率有所降低，将来有待于进一步完善我们的研究。对于临床上遇到的实性SPN患者，一定要客观判断，早期处理。
